# Post-traumatic stress symptoms experienced by healthcare workers in Lebanon four months following Beirut’s ammonium nitrate explosion: a survey-based study

**DOI:** 10.1186/s13690-022-00911-5

**Published:** 2022-06-17

**Authors:** Elie Bou Sanayeh, Carolla El Chamieh, Marie Christelle Saade, Rami George Maalouf, Maya Bizri

**Affiliations:** 1 Department of Internal Medicine, American University of Beirut Medical Center, Beirut, Lebanon; 2 Faculty of Medicine, Sorbonne University, Paris, France; 3 Department of Psychiatry, American University of Beirut Medical Center, Beirut, Lebanon

**Keywords:** Post-traumatic stress disorder symptoms, Beirut blast, Healthcare workers

## Abstract

**Background:**

On August 4, 2020, Lebanon faced one of the deadliest mass casualty explosions the world has witnessed during the twenty-first century. The human and emotional tolls were heavy on attending physicians, clinical fellows, residents, interns, medical students, and registered nurses, who were working in dramatic conditions, triaging, and treating thousands of blast-related casualties. We evaluated the risk of developing post-traumatic stress disorder symptoms (PTSS), among these healthcare workers (HCWs) from different Lebanese hospitals.

**Methods:**

This is a multicentered, cross-sectional study that was conducted in December 2020, using an online questionnaire that evaluated the risk of developing PTSS based on the validated self-reported PTSD-Checklist for DSM-V (PCL-5). We also explored possible correlates with the participants’ socio-demographic characteristics, job profile, mental health, and blast-related events.

**Results:**

Out of 519 participants, 44% were at high risk of developing PTSS following Beirut-blast. Nurses, attending physicians, fellows, and participants who are older in age, married, or working at specific hospitals, were at a higher risk. Those identified at higher risk of PTSS were surgeons, anesthesiologists, emergency medicine doctors, or radiologists; and they were more likely to be willing to migrate; having a prior history of psychiatric medication intake for PTSD treatment, a prior history of PTSD, or a personal history of seeking mental health service. At last, the latter two parameters as well as the number of examined injuries, severe home damage, and testing positive for the COVID-19 virus during the two weeks’ period that followed the blast were found to be predictors for the development of PTSS.

**Conclusion:**

Lebanese in-hospital HCWs were found to be at a high risk of developing PTSS following the Beirut-Blast, thus we recommend public health authorities to provide adequate resources to avoid the emergence of mental illnesses among these rescuers.

## Background

On August 4, 2020, Lebanon faced one of the deadliest mass casualty explosions the world has witnessed during the twenty-first century [1–3]. A harborfront explosion devastated the Lebanese city of Beirut [1, 2]. Tons of ammonium-nitrate exploded, thousands were injured, and hundreds were killed right away [1–3]. Citizens faced a sharp decline in healthcare provision in the perimeter of the explosion with many of the health-personnel being injured or killed, and several major hospitals being destroyed and no longer functional [1]. This event imposed a debilitating burden to the community, hospital system, and healthcare providers [4]. It superimposed many heavy challenges Lebanon was already facing noting the ongoing financial crisis, the increase in extreme poverty rate, the laid of health personnel due to economic constraints, and the significant increase in COrona VIrus Disease-19 (COVID-19) cases [2, 5]. On that day, in-hospital disaster-rescuers were exhausted while working in dramatic conditions, triaging and treating injured citizens, and facing nearly 200 confirmed fatalities [1]. Approximately 6500 citizens were moderately to critically injured of which around 1300 underwent surgery [4]. More than 145000 citizens required psychological support of which around 24000 in need for urgent mental care [4]. Thus, the psychological distress of Lebanese citizens is to be taken in account, especially when it comes to healthcare workers (HCWs).

In many previous trauma-outcome studies on disaster-rescuers, psychiatric impairment has proven to generate a greater impact on quality of life than physical injury. Post-traumatic stress disorder (PTSD) is the most prevalent disaster-related psychiatric sequalae [6]. According to the Diagnostic and Statistical Manual of Mental Disorders-5^th^ edition (DSM-5), PTSD is defined as a delayed and lasting mental health condition that occurs at least one month following the exposure to a life-threatening event, serious injury, natural disaster, or sexual violence [7]. It is characterized by standard intrusive manifestations, persistent stimuli avoidance, negative alterations in cognitions or mood, as well as hyperarousal of certain physiological functions [7]. While the lifetime prevalence in the general population is between 2.1% [8] and 9.2% [9], the incidence can be as high as 58.2% among direct disaster-victims, such as following an earthquake [10]. Experimental studies have documented higher rates among HCWs, with a prevalence ranging from 8% to 40.7% when facing extremely stressful conditions, such as pandemics [11]. Multiple risk factors for PTSD have been identified such as younger age, female gender, being unmarried, low household income, being unemployed, history of psychiatric illnesses, intensity of the traumatic event, lack of experience in handling stressful conditions and the severity of encountered injuries [9, 10].

Early screening and intervention proved to help victims in maintaining their mental health [6]. However, to date, no studies have been published in Lebanon, addressing the psychological impact of Beirut-blast on HCWs. The objective of this study was to evaluate the risk of developing post-traumatic stress symptoms (PTSS) post-Beirut’s ammonium-nitrate explosion, in attending physicians, clinical fellows (post-graduation year >3: PGY>3), residents (PGY-2 and -3), interns (PGY-1), pre-final- (third) and final- (fourth) year medical students, and registered nurses, working in different Lebanese hospitals. We also explored possible correlates with participants’ socio-demographic characteristics, job profile, mental health, and blast-related events.

## Methods 

### Study design

This is a multicentered, cross-sectional, study that was conducted in December 2020. An electronic consent was sent to participants and participation was entirely on a voluntary basis. Participants had the right to withdraw their consent or discontinue participation at any time without penalty. The institutional review board at the American University of Beirut has approved the study design (IRB number: SBS-2020–0449).

### Sampling procedure and data collection

During data collection, Lebanon was undergoing a nationwide lockdown. Subsequently, in order to enroll potential participants, an online questionnaire was developed on the electronic platform of LimeSurvey (LimeSurvey GmbH, Hamburg, Germany). It was sent to registered nurses via the Lebanese order of nurses, and via WhatsApp messenger (Facebook Inc.) to a representative in each hospital who subsequently diffused it to corresponding attending physicians, clinical fellows, residents, and interns from all specialties as well as, third- and fourth-year medical students. Non-medical staff was not included.

### Questionnaire

The main study-tool was a self-reported questionnaire (see additional file 1) written in English which is, to varying degrees, the official language of medical instruction in Lebanon. It started with a quick introductory note briefing the study objectives and ensuring response confidentiality and anonymity, followed by the consent form. The questionnaire consisted of 42 closed-ended questions divided into three sections:

1. Sociodemographic characteristics (age, gender, marital status, residency location), job profile (profession, specialty, institutional affiliation, personal income per month) and mental health (personal history of PTSD, seeking mental health service and intake of psychiatric medications).
2. Questions about the events that happened on that day: location at the moment of the blast, its impact on applicant’s health, catching the COVID-19 virus following the blast, home and work status, injury or loss of a relative, friend or co-worker, number of examined casualties, degree of satisfaction of the accomplished work, and willingness to migrate.
3. PTSD Checklist for DSM-5 (PCL-5) which is a comprehensive, self-reported, and validated scale in English [12]. By assessing PTSD, based on the DSM-5 criteria, it aims to assess the risk of developing PTSS. It consists of 20 items, rated on a 5-point Likert-type scale with scores ranging from 0 “Not at all” to 4 “Extremely” [12]. The overall score can range from 0 to 80 with a total cut-off score of ≥ 33 indicating a high risk of developing PTSS, thus the need for further evaluation by a specialist [12].

### Statistical analysis

The internal reliability of the PCL-5 scale was checked, and a Cronbach’s alpha of 0.94 was recorded. We also confirmed the internal structure of the scale by a Confirmatory Factor Analysis (CFA) using the lavaan package of R software. Several goodness‐of‐fit indicators were reported in Table 1: the Relative chi‐square (χ2/df) (cut-off values: < 2–5), the Root Mean Square Error of Approximation (excellent and acceptable fit are considered for values < 0.05 and < 0.11, respectively), the standardized root mean square residuals (acceptable values are <  = 0.08), the Tucker Lewis Index, and the Comparative Fit Index (acceptable values are ≥ 0.95). The value of χ2/df has a low sensitivity to sample size and may be used as an index of goodness-o-fit [13].

**Table 1:** Confirmatory Factor Analysis of the PCL-5 scale

χ2/df	CFI	TLI	SRMSR	RMSEA
757/175 = 4.3	0.993	0.992	0.058	0.07

χ2*/df* relative chi‐square, *CFI* Comparative Fit Index, *TLI* Tucker-Lewis index, *SRMSR* standardized root mean square residuals, *RMSEA* root mean square error of approximation

Continuous variables are presented as mean (standard deviation) and were compared between groups using Wilcoxon rank sum test. Categorical variables are presented as frequency (percentage) and were compared between groups using Chi-square test or Fisher’s exact test accordingly. Multiple logistic regression was carried out to determine predictors of PTSS, so the assessed risk of PTSS was used as the dependent variable. *P*-values lower than 0.05 were considered as indicating a significant association. Analyses were performed using R statistical software, version 4.0.2.

### Sample size

A minimal sample size of 384 was required according to the following formula [14]: $\mathrm{n}=\frac{{Z}^{2}*`P*(1-P)}{{d}^{2}}$, where n is the sample size, Z = 1.96 is the statistic corresponding to a 95% level of confidence, *P* = 50% is the expected prevalence (as there are no comparable studies in Lebanon) and d = 5% is the precision.

## Results

In total 519 participants completed the questionnaire and were included in the data analysis.

### Participants’ characteristics

Participants’ sociodemographic and personal characteristics are summarized in Table 2. The mean age of participants was 30 years, with 73% of them being ≤ 30 years. 57% were females, 26.5% were married, and 60% living in Beirut. Concerning participants’ job-related information: 26% were nurses, 19% residents and 18% medical students. They were mainly from medical specialties (29.5%), surgical specialties (29%), and anesthesia/emergency medicine (14%). The majority (59%) were middle-income earners (between 1.000.000 and 2.999.999 Lebanese pounds (LBP) per month) and 75.3% were willing to migrate. Concerning prior psychiatric illnesses, 12.5% were seeking help from a professional mental health provider and 7.7% were on psychiatric medications, out of which, respectively 31% and 25% had a history of PTSD. Overall, 11.2% had a personal history of PTSD.

**Table 2:** Participants’ sociodemographic/personal characteristics, and their associations with a higher risk of developing PTSS four months following Beirut’s blast

Characteristics	Total *N* = 519^a^	No Risk^a^ *n* = 291 (56%)	Risk of PTSS^a^ *n* = 228 (44%)	*p*-value^b^
**Gender**				0·28
Female	297 (57%)	156 (55%)	141 (60%)	
Male	222 (43%)	128 (45%)	94 (40%)	
**Age**				**0·002**
≤ 30 years	380 (73%)	228 (80%)	152 (64·5%)	
> 30 years	139 (27%)	63 (20%)	76 (35·5%)	
**Married**				**0·01**
Yes	138 (26·5%)	65 (22·5%)	73 (32%)	
No (single, divorced, widowed)	381 (73·5%)	226 (77·5%)	155 (68%)	
**Residency location**				0·98
Beirut	310 (60%)	169 (58%)	141 (62%)	
Outside Beirut	209 (40%)	115 (42%)	94 (38%)	
**Personal income per month**				0·19
< 1·000·000 LBP	113 (22%)	72 (25%)	41 (18%)	
1·000·000 LBP—2·999·999 LBP	305 (59%)	171 (59%)	134 (59%)	
≥ 3·000·000 LBP	101 (19%)	48 (16%)	53 (23%)	
**Job profile**				**0·002**
Attending physician	74 (14%)	33 (11%)	41 (18%)	
Clinical fellow (PGY > 3)	45 (9%)	23 (8%)	22 (10%)	
Resident (PGY-2 and PGY-3)	100 (19%)	67 (23%)	33 (14%)	
Intern (PGY-1)	72 (14%)	42 (15%)	30 (13%)	
Medical student	94 (18%)	60 (22%)	34 (13%)	
Registered nurse	134 (26%)	59 (21%)	75 (32%)	
**Specialty (if physician, fellow, resident or intern) (*n* = 291)**		*n* = 151	*n* = 140	**0·005**
Internal Medicine	86 (29·5%)	47 (31%)	39 (28%)	
Surgical specialties	84 (29%)	40 (26%)	44 (31%)	
Anesthesia + Emergency medicine	40 (14%)	18 (12%)	22 (16%)	
Pediatrics	24 (8%)	16 (11%)	8 (6%)	
Psychiatry	8 (3%)	8 (5%)	0 (0%)	
Radiology	10 (3%)	4 (3%)	6 (4%)	
Other	39 (13·5%)	18 (12%)	21 (15%)	
**Hospital working at:**				**0·001**
American University of Beirut Medical Center (AUBMC)	174 (33·5%)	112 (38·5%)	62 (27%)	
Hôpital Notre Dame des Secours (NDS)	92 (17·7%)	57 (19·5%)	35 (15·5%)	
Hôtel-Dieu de France (HDF)	65 (12·5%)	40 (14%)	25 (11%)	
Saint George Hospital University Medical Center	33 (6·4%)	16 (5·5%)	17 (7·5%)	
LAU Medical Center-Rizk Hospital	29 (5·6%)	14 (5%)	15 (6·5%)	
Lebanese Hospital Geitaoui (LHG)	13 (2·5%)	4 (1%)	9 (4%)	
Mount Lebanon Hospital (MLH)	8 (1·5%)	2 (0·5%)	6 (2·5%)	
Other	105 (20·3%)	46 (16%)	59 (26%)	
**Willingness to migrate**	391 (75·3%)	205 (70·5%)	186 (81·5%)	**0·004**
**Personal history of PTSD**	58 (11·2%)	18 (6%)	40 (17·5%)	** < 0·001**
**Overall seeking a professional mental health provider**	65 (12·5%)	22 (7·5%)	43 (19%)	** < 0·001**
**Seeking a professional mental health provider**				**0·006**
With personal history of PTSD	20 (31%)	2 (9%)	18 (42%)	
Without personal history of PTSD	45 (69%)	20 (91%)	25 (58%)	
**Overall psychiatric medications intake**	40 (7·7%)	20 (7%)	20 (9%)	0·4
**On any psychiatric medications**				**0·003**
With personal history of PTSD	10 (25%)	1 (5%)	9 (45%)	
Without personal history of PTSD	30 (75%)	19 (95%)	11 (55%)	

*p*-values in bold are considered significant

^a^Statistics presented: n (%)

^b^Statistical tests performed: chi-square test of independence; Wilcoxon rank-sum test; Fisher's exact test

*AUBMC* American University of Beirut Medical Center, *HDF* Hôtel-Dieu de France, *LBP* Lebanese Pound, *LHG* Lebanese Hospital Geitaoui, *MLH* Mount Lebanon Hospital, *NDS* Hôpital Notre Dame des Secours, *PGY* Post-Graduation Year, *PTSD* Post-Traumatic Stress Disorder, *PTSS* Post-Traumatic Stress Symptoms

### Participants’ characteristics in association with the high risk of developing PTSS

Table 2 also summarizes the association between the abovementioned characteristics and the risk of developing PTSS. A high risk was significantly associated with older age (> 30 years old, *p* = 0.002), marital status (being married, *p* = 0.01), job profile (nurses, attending physicians and fellows, *p* = 0.002), specialties (surgical specialties, anesthesia, emergency medicine, and radiology, *p* = 0.005), and the hospital in which participants work (Saint George hospital, Rizk hospital, Lebanese Hospital Geitaoui- LHG, and Mount Lebanon Hospital-MLH, *p* = 0.001). It is important to note that 56% of nurses, 55.4% of physicians, 48.8% of fellows, 41.6% of interns, 36.1% of medical students and 33% of residents had a high risk of developing PTSS following the blast. In addition, willing to migrate (81.5% vs. 70.5%, *p* = 0.004), having a personal history of PTSD (17.5% vs. 6%, *p* < 0.001) and seeking help from a mental health provider (19% vs. 7.5%, *p* < 0.001) were associated with developing PTSS. By further dividing them, seeking a professional help or being on psychiatric medications especially in someone with a personal history of PTSD were considered significantly associated with a higher risk of developing PTSS (respectively *p* = 0.006 and *p* = 0.003).

### Events that happened on the day of the blast

Table 3 summarizes the events that happened on the day of the blast and their associations with a higher risk of developing PSTD. At the time of the blast, 45.8% of surveyed participants were at home, while 34.2% were in the hospital. Following the blast, 17% had their homes severely damaged, 10% got injured and 2.5% lost their work. Participants have encountered a mean of 19 injured patients, and three blast-related deaths, making 70% of them proud of their work. Also, 61% had a relative, friend or co-worker who got injured, and 19% had one of them dead secondary to the blast. During the two weeks’ period that followed the blast, 11% have tested positive for the COVID-19 virus. (Table 3).

**Table 3:** Events that happened on the day of the blast and their associations with having a high risk of developing PTSS four months following Beirut’s blast

Characteristics	Total *N* = 519^a^	No Risk^a^ *n* = 291 (56%)	Risk of PTSS^a^ *n* = 228 (44%)	*p*-value^b^
Got injured the day of the blast	51 (10%)	12 (4%)	39 (17%)	** < 0·001**
Loss of a relative, a friend or a co-worker the day of the blast	100 (19%)	35 (12%)	65 (28·5%)	** < 0·001**
Injury of a relative, a friend or a co-worker the day of the blast	317 (61%)	171 (59%)	146 (64%)	0·15
Tested positive for COVID-19 virus during the two weeks’ period that followed the blast	57 (11%)	9 (3%)	48 (21%)	** < 0·001**
Home severely damaged by the blast	89 (17%)	32 (11%)	57 (25%)	** < 0·001**
Work lost following the blast	13 (2·5%)	3 (1%)	10 (4%)	0·03
Number of examined injuries during the 24 h that followed the blast	19 (19)	14 (15)	25 (22)	** < 0·001**
Number of encountered blast-related deaths during the 24 h that followed the blast	3 (3)	2 (3)	4 (5)	** < 0·001**
Proud of your work on that day	364 (70%)	205 (70·5%)	159 (70%)	0·86
Participant’s location at the time of the blast				0·1
At home	238 (45·8%)	129 (44·3%)	109 (47·8%)	
At the hospital	177 (34·2%)	104 (35·7%)	73 (32%)	
On the road (Beirut area)	57 (11%)	26 (8·9%)	31 (13·6%)	
Other	47 (9%)	32 (11%)	15 (6·6%)	

*p*-values in bold are considered significant

^a^Statistics presented: n (%); Mean (SD)

^b^Statistical tests performed: chi-square test of independence; Wilcoxon rank-sum test

PTSS: Post-traumatic Stress Symptoms; COVID-19: COrona VIrus Disease-19

### Events that happened on the day of the blast in association to the high risk of developing PTSS

HCWs who got injured by the blast (*p* < 0.001), lost a relative, a friend or a co-worker on that day (*p* < 0.001), tested positive for COVID-19 during the two weeks’ period that followed the blast (*p* < 0.001), had their home severely damaged (*p* < 0.001), lost their work (*p* = 0.03), or have encountered a relatively higher number of injured patients (*p* < 0.001) or deaths related to the blast (*p* < 0.001) were all significantly associated with a higher risk of developing PTSS. (Table 3).

### PCL-5 results

Table 4 summarizes the distribution of participants’ responses on the PCL-5 checklist. Out of 519 participants, 228 had a total score of ≥ 33 on the PCL-5 questionnaire. Thus, 44% of our applicants are considered at high risk of developing PTSS related to the blast.

**Table 4:** Distribution of the answers on the PTSD Checklist (PCL-5) scale

**PTSD checklist (PCL-5)**^**1**^ **(*N* = 519)**	**Not at all (0)** ***n (%)***	**A little bit (1)** ***n (%)***	**Moderately (2)** ***n (%)***	**Quite a bit (3)** ***n (%)***	**Extremely (4)** ***n (%)***
Repeated, disturbing, and unwanted memories of the blast?	104 (20%)	107 (21%)	98 (19%)	117 (23%)	93 (18%)
Repeated, disturbing dreams of the blast?	211 (41%)	100 (19%)	75 (14%)	69 (13%)	64 (12%)
Suddenly feeling or acting as if the blast was happening again?	194 (37%)	97 (19%)	80 (15%)	74 (14%)	74 (14%)
Feeling very upset when something reminded you of the blast?	72 (14%)	97 (19%)	104 (20%)	109 (21%)	137 (26%)
Having strong physical reactions when something reminded you of the blast (such as heart pounding, trouble breathing, sweating)?	192 (37%)	112 (22%)	71 (14%)	75 (14%)	69 (13%)
Avoiding memories, thoughts, or feelings related to the blast?	132 (25%)	100 (19%)	91 (18%)	96 (18%)	100 (19%)
Avoiding external reminders of the blast (people, places, conversations, activities, objects, or situations)?	169 (33%)	95 (18%)	88 (17%)	86 (17%)	81 (16%)
Trouble remembering important parts of the blast or what happened after it?	247 (48%)	74 (14%)	66 (13%)	74 (14%)	58 (11%)
Having strong negative beliefs about yourself, other people, or the world	193 (37%)	95 (18%)	62 (12%)	85 (16%)	84 (16%)
Blaming yourself or someone else for the blast or what happened after it?	253 (49%)	80 (15%)	60 (12%)	73 (14%)	53 (10%)
Having strong negative feelings such as fear, horror, anger, guilt, or shame?	157 (30%)	104 (20%)	100 (19%)	78 (15%)	80 (16%)
Loss of interest in activities that you used to enjoy?	140 (27%)	108 (21%)	93 (18%)	100 (19%)	78 (15%)
Feeling distant or cut off from other people?	146 (28%)	119 (23%)	79 (15%)	94 (18%)	81 (16%)
Trouble experiencing positive feelings	164 (32%)	115 (22%)	83 (16%)	88 (17%)	69 (13%)
Irritable behavior, angry outbursts, or acting aggressively?	149 (29%)	101 (19%)	111 (21%)	66 (13%)	92 (18%)
Taking too many risks or doing things that could cause you harm?	286 (55%)	72 (14%)	61 (12%)	55 (11%)	45 (8%)
Being “superalert” or watchful or on guard?	138 (27%)	117 (23%)	101 (19%)	77 (15%)	86 (16%)
Feeling jumpy or easily startled?	164 (32%)	116 (22%)	94 (18%)	63 (12%)	82 (16%)
Having difficulty concentrating?	131 (25%)	108 (21%)	120 (23%)	92 (18%)	68 (13%)
Trouble falling or staying asleep?	169 (33%)	120 (23%)	79 (15%)	77 (15%)	74 (14%)

High risk of developing PTSS related to Beirut-blast if total score is ≥ 33

*PCL-5* PTSD Checklist for DSM-5, *PTSD* Post-traumatic Stress Disorder, *PTSS* Post-traumatic Stress Symptoms

### Willingness to migrate

**Fig. 1:**
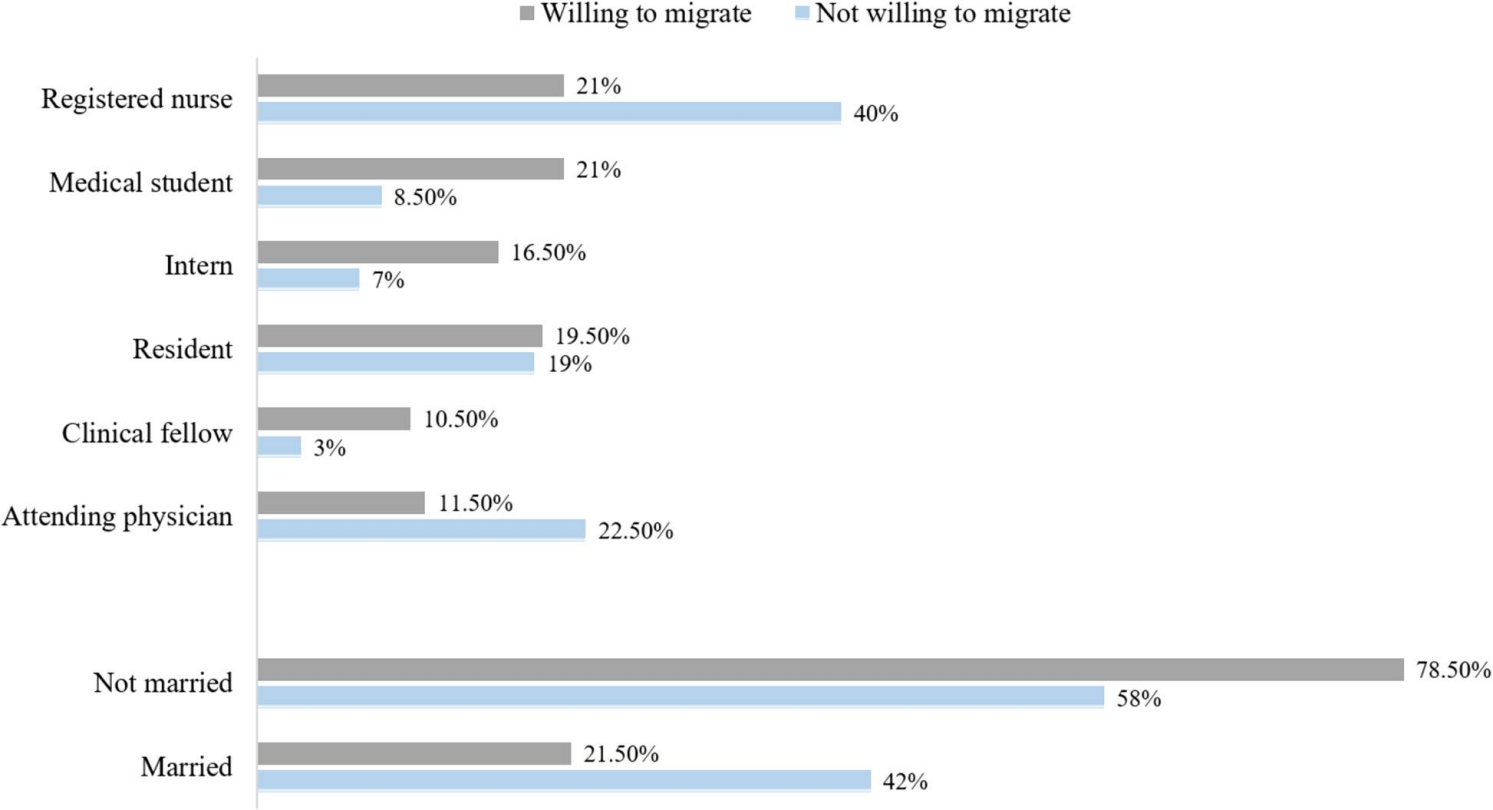
Significant association (*p* < 0.001) between willingness to migrate and job profile/marital status

Among the 75.3% who are willing to migrate, significantly (*p* < 0.001) those who are married had lower rates (60.8% vs 80%). As well, being a fellow, intern, student, or resident was significantly associated (*p* < 0.001) to this parameter with respectively 91%, 88.8%, 87%, and 76% of them seriously considering it, in contrast to the 61% reported in nurses and 60% in physicians. (Fig. 1).

### Predictors of PTSS

**Fig. 2:**
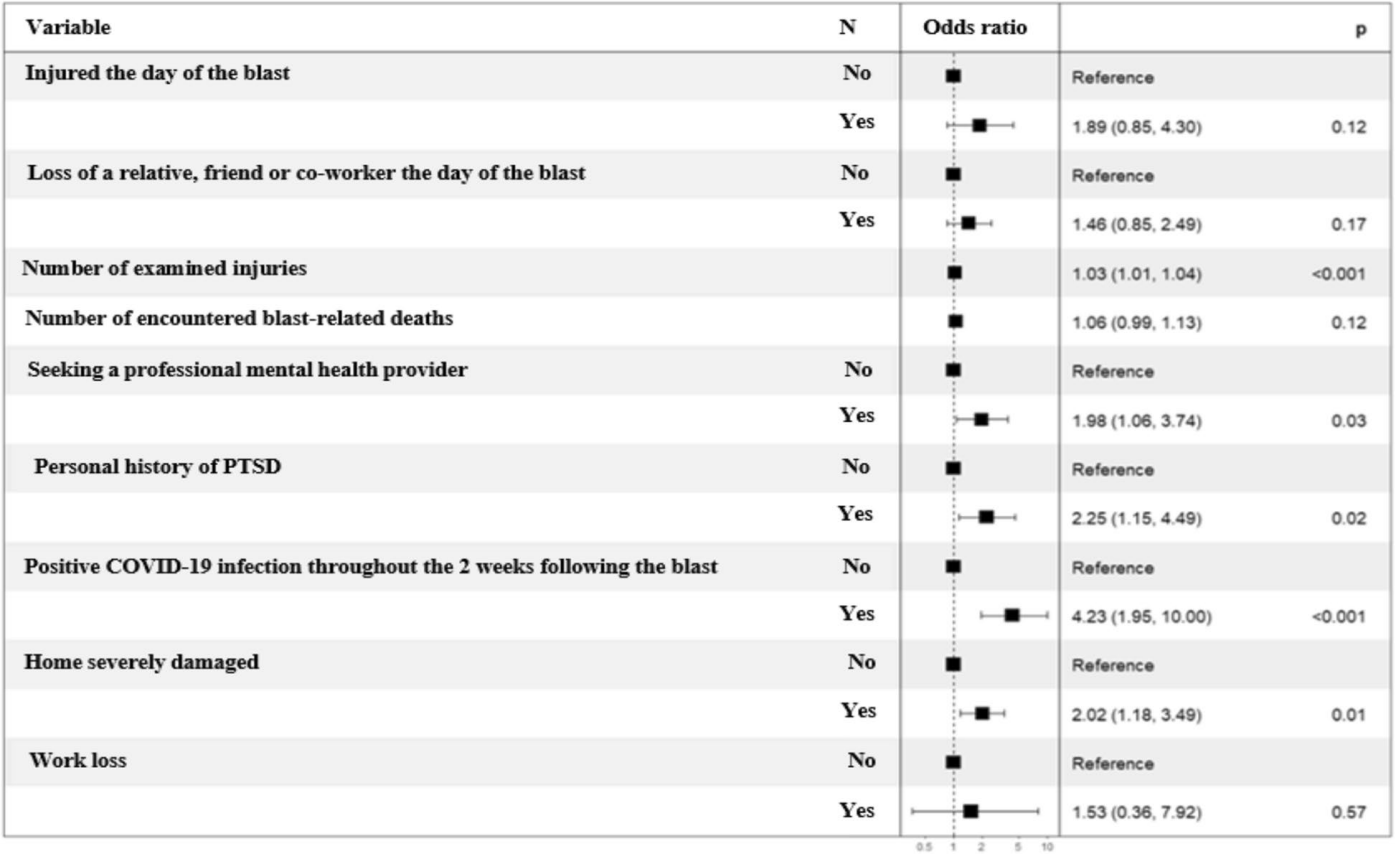
Multiple logistic regression analysis of factors associated with the risk of PTSS four months following Beirut’s ammonium nitrate explosion. Candidate variables entered: age, current marital status, job profile, specialty, hospital working at, personal history of PTSD, seeking a professional mental health provider, being on any psychiatric medications, being injured the day of the blast, injury, or death of a relative, a friend or a co-worker the day of the blast, testing positive for COVID-19 virus during the two weeks’ period that followed the blast, home severely damaged by the blast, loss of work, number of encountered injuries and blast-related deaths

The results of the logistic regression, adjusted to the variables retained by the model selection by elimination and then to the assessed risk of PTSS, are presented in Fig. 2. The loss of a relative, a friend or a co-worker, being injured the day of the blast, the number of encountered deaths, and work loss had no statistically significant effect on the risk of developing PTSS. However, the number of examined injuries slightly increased the risk (OR = 1*.*03; *p* < 0.001). Significantly, three variables have almost doubled the risk of PTSS which are: seeking a professional mental health provider (OR = 1.98, *p* = 0.03), severe home damage (OR = 2.02, *p* = 0.01), and a personal history of PTSD (OR = 2.25, *p* = 0.02). At last, participants who tested positive for the COVID-19 virus during the two weeks’ period that followed the blast had 4.23 times higher odds of developing PTSS (*p* < 0.001) than those who did not catch the virus.

## Discussion

To date, there has been no comprehensive studies to evaluate the risk of developing PTSS in “in-hospital HCWs” following the devastating Beirut-blast that occurred in August 2020. The CFA of the PCL-5 scale lends support to its validity as a measure of PTSS risk in the Lebanese population increasing the reliability of our results.

The widespread destruction after Beirut-blast has led to a high risk of developing PTSS in 44% of our surveyed population. These results align with the existing literature about the prevalence of PTSD after human-made disaster. In fact, in comparison to the literature, our findings were similar to the ones reported in a global meta-analysis on PTSD incidence among rescuers of the 9/11 World Trade Center attacks in the USA (42%) [15]. However, it was higher than the projected lifetime risk expected for the general adult population from different countries [8, 9]. It was also higher than the noted prevalence in Intensive Care Unit workers (8% to 30%) [16], in HCWs following several pandemics (16.7% to 40%) [8, 11, 17], and even in British and American veterans returning from the Vietnam and Iraq Wars (2% to 17%) [18]. As per Bromet et al., disaster-related PTSD is higher in man-made disasters compared to natural disasters, and it significantly correlates with higher education, serious injury or death of someone close, being displaced by the disaster and pre-existing vulnerabilities [19]. Our statistical results, which are on the upper limit of the usual range of disaster-related PTSD prevalence confirm these findings. Beirut-blast was a man-made disaster, our Lebanese population suffers from pre-existing vulnerabilities and since we targeted HCWs only, our population is a highly educated one. The death of a close person and being displaced from its own place were both correlated to higher risk of developing PTSS.

These results can also be the consequence of the sudden impact imposed by this unexpected event, putting HCWs in a stressful work-related situation. They were forced to treat thousands of critically injured patients and manage severely traumatized people [2]. They experienced war-like scenes, witnessing death, explaining to families the challenging expectations of their beloved patients, and confronting an imbalance between actual needs and available resources [2].

Furthermore, our participants were found to be heterogeneous regarding sociodemographic characteristics, and their association with the risk of developing PTSS. Those who were married and still living with their partner had a higher risk possibly because they were older in age thus previously exposed to many traumatizing events [20, 21], or because they were afraid that their children got injured [16]. A recent meta-analysis [16] revealed contradictory data with some studies reporting the same positive association [22], however, others revealing that those who were unmarried were more adversely affected [15, 16, 20]. Also, no difference was noted between gender. It may seem counterintuitive, given that female gender has been mostly linked to higher rates of PTSD [15, 16]. However, a meta-analysis of 11 Lebanese studies revealed that gender was not explanatory of PTSD [23] comparably to what was reported in American studies done on female military and police officers [20, 24]. This could be possibly explained by the minimization of gender-related variations following the rigorous selection and training.

Concerning the occupational role, nurses were at the higher risk of developing PTSS, followed by attending physicians and fellows. In contrast, being a medical student, intern or resident was not associated. These findings are similar to those of previous studies that highlighted the occupational role as a major risk factor for PTSS [16, 25], with nurses being more likely to develop distress than physicians given that they are exposed to more patients [26]. This could be explained by the fact that nurses do not have defined national work-hour policies [26]. They are continuously challenged physically and psychologically by long working hours, and unplanned fluctuations in shift lengths because of unpredictable staffing changes [26].

As well, in our study, all categories have scored higher than what was reported in the literature (56% >16.8% to 18%(1) [8, 27] in nurses, 55.4% >15.8% to 16.8% [28, 29] in physicians, 48.8% to 33% >14% to 23% [17, 30] in fellows, interns and residents, and 36.1% >5.2% to 23.5% [31, 32] in medical students). As abovementioned, physicians and fellows also scored higher than their younger colleagues on the PCL-5. In fact, participants that were older than 30 years of age were found to be at a higher risk of developing PTSS. These findings are conceivable in Lebanon given that this age category was previously exposed to the civil war and internal conflicts that this country has been plagued by for decades (1975-2006) [23]. Effectively, being exposed to repetitive disasters and being the decision-maker in distressing situations are all known risk factors for PTSS [15, 20].

Besides occupation, the risk significantly varied across medical specialties. Surgical specialties, anesthesia, and emergency medicine were at the highest risk. To varying degrees, work-related stressors are identified, among all medical fields, but these specialties in their role as first responders are repetitively challenged by unpredictable serious casualties [16, 17, 20, 30]. On the day of the blast, they faced the suffering of high-acuity victims, on whom life-saving interventions had to be done. Our findings were comparable to prior studies [28], but came in contrast with one previous study conducted by Jackson and colleagues who reported no statistical difference in PTSS prevalence between specialties [30]. Regardless of their occupational role or specialty, HCWs working at Saint George hospital, Rizk hospital, and LHG showed a higher risk for developing PTSS. This could be easily explained by the fact that these hospitals are in close perimeter to the blast and were considered among the seven most affected hospitals [4]. This fact is consistent with prior reviews that identified the amount of trauma exposure as a primary risk factor for PTSS [16, 23].

An additional examined parameter that showed significant association with the risk of developing PTSS was willingness to migrate. This willingness was significantly associated with the marital status and job profile. Those who are single or not fully settled (fellows, interns, students, or residents) were considering migration the most, possibly because they still have the freedom to seek better opportunities abroad. In fact, Lebanon has always been known by the “culture of emigration” among physicians in training.

Furthermore, a variety of factors have been associated with the increased risk for PTSS. According to Kessler and colleagues, traumas can be divided into multiple categories [21]. For purposes of analysis, evaluated blast-related parameters that were associated to a higher risk of developing PTSS in our study, were allocated into four of these categories. First, when it comes to physical trauma, two associations were found: being injured the day of the blast and testing positive for COVID-19 during the two weeks’ period that followed the blast. In fact, traumas involving violence with subsequent physical pain have been identified as negative consistent reminders of the trauma [15, 21, 23]. In addition, the night of the blast HCWs were particularly exposed to the threat of COVID-19 transmission due to the lack of adequate precaution measures. Our statistical results showed that testing positive for COVID-19 is a predictor for developing PTSS (OR=4.23). In the current pandemic, getting the virus has been already described as a relevant risk factor for PTSS development [16] given the unpredictable course of the disease and high mortality rates [12]. Following Beirut-blast, many factors were added and amplified the risk of PTSS in COVID-19 infected HCWs. In a time where emotional encouragement and social support are needed most [33], self-isolation and social stigmatization of HCWs deeply affected the coping mechanisms of infected individuals [16]. Second, regarding trauma of beloved ones, the loss of a relative, a friend or a co-worker was an associated factor. Among all lifetime stressful experiences, the unexpected death of a loved one has been previously reported, as having the highest traumatic burden on one’s life [34]. Third, in view of traumas related to financial loss, loosing work or home were established as associations. Notably, homelessness was also a predictor for PTSS (OR=2.02), which is consistent with Goodman and colleagues’ results [35]. However, the multiple logistic regression analysis showed that work-loss had no predicting effect on developing PTSS in contrast to what was previously stated following the 9/11 attacks [36].

Additionally, regarding traumas that happen to other people, the number of examined injuries was not only an associated parameter but also a predicting factor for the development of PTSS. This could be explained by the fact that the unpredicted flow of casualties required life-saving interventions and heightened the decision-making burden on HCWs [2].

To sum up, our associated parameters were comparable to those reported in a prior Lebanese systematic review, with the most devastating traumas among the Lebanese population being the loss of a loved one, injury to self or others, and home-loss [23].

Finally, an additional major consideration is the psychiatric history, given that a previous history of PTSD, seeking a mental health service for this condition and a history of medication intake for PTSD treatment, were found to be associated with a higher risk of developing PTSS following Beirut-blast. Among the abovementioned associations and consistent with previous data [37], a history of PTSD (OR=2.25) or seeking a mental health provider (OR=1.98) were found to be predictors for the development of PTSS. Prior studies have stressed the presence of previous PTSS as a predictor for its recurrence [16, 22]. Development of PTSS in someone with a prior history of the illness could be considered as a continuation of a chronic, unremitted event, with a preexisting vulnerability to subsequent traumas [37].

### Clinical implications

The current study provides many valuable contributions to the literature. This is the first study to shed light on the psychological impact of Beirut-blast on HCWs. Our findings constitute a first step toward encouraging public health authorities to support in-hospital HCWs. This population has a pivotal role in our society and experience an extremely high burden during man-made and natural disasters.

It would be optimal to prepare HCWs mentally for crisis management and create a protective and supportive working environment. Indeed, HCWs need to be trained to cope with traumatic events through evidence-based anticipatory methods and educated to support their colleagues after stressful events. Moreover, organizing psychological debriefing sessions could be helpful by encouraging HCWs to share their emotions and experiences. These interventions should be applied as soon as possible after the disaster. For instance, several measures should also be implemented to minimize the impact of such scenarios on the psychological status of HCWs, such as sleep hygiene, flexible work schedules, sports, and cultural experiences facilities (access to films, concerts). We also emphasize on the need for a rigorous systematic screening of all exposed HCWs for emerging mental disorders following large traumatic events. Short questionnaires assessing HCW’s mental health should be used repeatedly - subjects with alarming scores must be referred for further professional psychological assessment. Hence the importance of implementing free psychological consultations. These major adjustments will not only boost HCWs’ morale but will be beneficial for society in the long run.

Some efforts were mobilized to help the Lebanese general population affected by the blast.

For instance, a hotline was created for people in mental distress; some centers offered free consultations with specialists for those who were physically, financially or morally affected by the blast; several NGOs offered financial and moral support for people who lost their homes or families. Although HCWs experienced an enormous burden, they did not receive the right support at the right time. We believe that the majority were underdiagnosed and did not seek care for their condition. Thus, we believe that reporting these results may shed the light on the importance of taking into account the mental health of HCWS in the aftermath of a disaster scenario.

### Limitations

The interpretation of our results should take into consideration some limitations. First, given that we are using a self-reported questionnaire that might be influenced by respondent’s level of interest, the possibility of self-reporting bias cannot be ruled out. Second, the study was conducted four months following the blast which could also lead to recall bias. As well, we did not assess the pandemic-related psychological distress given its proven impact on HCWs mental health in Lebanon 31. Furthermore, the Lebanese population and specifically the HCWs have faced multiple traumatic events throughout the years such as civil war, aggressions, ongoing economic crisis and political instability. We controlled for this confounder by using questions in our survey that are very specific to the Beirut Blast. However, we believe that this could have affected our results and remain a limitation to our study. At last, the study used a cross-sectional design, which hinders it from determining causality. However, despite the study limitations, our findings helped in quantifying the toll that Beirut-blast has taken on in-hospital HCWs who were involved in rescuing injured citizens in dramatic conditions.

## Conclusion

Beirut-blast is one of the most powerful explosions the world has ever witnessed. While taking into consideration possible predicting factors, it was reported in this study that Lebanese in-hospital HCWs who experienced this horrendous traumatic event are at a high risk of developing PTSS. It is the duty of public health caregivers to develop a systematic screening plan with pre-defined management algorithms, to avoid the emergence of mental illnesses among HCWs. Meanwhile, further studies should be conducted to assess more precisely the prevalence of PTSS and other potential mental health conditions among Lebanese in-hospital rescuers.

## Data Availability

All data generated or analyzed during this study are not publicly available to maintain the privacy of the individuals’ identities. The dataset supporting the conclusions is available upon request to the corresponding author.
